# Evaluating the effects of lymphoedema management strategies on functional status and health-related quality of life following treatment for head and neck cancer: Protocol for a systematic review

**DOI:** 10.1371/journal.pone.0297757

**Published:** 2024-02-02

**Authors:** Lauren J. Mullan, Nicole E. Blackburn, Jill Lorimer, Cherith J. Semple

**Affiliations:** 1 School of Nursing, Institute of Nursing and Health Research, Ulster University, Belfast, United Kingdom; 2 School of Health Sciences, Institute of Nursing and Health Research, Ulster University, Londonderry, United Kingdom; 3 Physiotherapy Department, Cancer Centre, Belfast Health and Social Care Trust, Belfast, United Kingdom; 4 Cancer Services, South Eastern Health and Social Care Trust, Belfast, United Kingdom; Xiamen University - Malaysia Campus: Xiamen University - Malaysia, MALAYSIA

## Abstract

**Introduction/Background:**

Patients living with and after head and neck cancer often experience treatment-related consequences. Head and neck lymphoedema can be described as a common chronic side effect of head and neck cancer and recognised as a contributing factor to impairment of functional status, symptom burden and health-related quality of life. The effects of head and neck lymphoedema can limit patients’ involvement in daily activities and alter their appearance, increasing symptom burden and negatively affecting health-related quality of life.

**Objective:**

The protocol outlines the rationale and aims for the systematic review. The main aim of the systematic review is to identify and systematically synthesise the literature on the effectiveness of head and neck lymphoedema management strategies, on both function status and health-related quality of life for head and neck cancer patients.

**Methods and analysis:**

This protocol will be conducted according to the PRISMA-P guidelines. Electronic databases will be systematically searched using MEDLINE via Ovid and PubMed, CINAHL, Cochrane Central Register of Controlled Trials and Scopus. Inclusion criteria will involve intervention studies for head and neck lymphoedema management, English language, and adult human participants following head and neck cancer. The software Covidence will be used to export, manage, and screen results. Risk of bias and quality will be assessed in included studies using the Cochrane Handbook of Systematic Reviews of Intervention risk of bias and GRADE tools. A meta-analysis will be performed if there are sufficient homogenous studies. Alternatively, a narrative synthesis will be completed on study findings.

**Ethics and dissemination:**

No ethical approval is required as the study does not involve patient and public involvement. The findings of the review will be disseminated in conferences and submitted for approval to be published in a peer-reviewed journal.

**Prospero registration number:**

CRD42022378417. (S1 Appendix).

## Introduction

Head and neck cancer (HNC) is stated as the 6^th^ most common cancer globally with more than 800,000 diagnoses recorded in 2018 [1]. In the United Kingdom (UK), 375,400 cancer cases were reported in 2018, of which 12,422 were a result of HNC [2]. While the incidence of this cancer has risen rapidly due to the association of the Human Papillomavirus (HPV) with oropharyngeal carcinoma, it is highly responsive to therapy, resulting in improved survival outcomes [3]. Progress continues to be made in detecting, diagnosing, and treating HNC, with a higher number of cancer survivors living longer with the associated cancer and treatment-related consequences [4].

Treatment for HNC consists of a multi-modal approach using a combination of surgery, radiotherapy, chemotherapy, and the promising emerging role of immunotherapy [5]. Despite these advances, the acute and chronic side effects of treatment have proven to have potential detrimental effects on HNC patients after treatment [6]. Head and neck lymphoedema (HNL) is described as being a common chronic side effect of HNC treatment, impacting functional status and health-related quality of life domains [7, 8].

Although HNL is a common consequence of treatment it is often under-recognised and therefore under-treated [6]. This life-altering health condition presents as an abnormal level of swelling and accumulation of protein rich fluid in patients’ interstitial spaces [9]. Swelling can commence when a patient is receiving treatment for HNC or during the survivorship phase [10]. A recent review highlighted that the prevalence of HNL varies because of methodological differences between studies [11]. It is estimated that the prevalence of HNL can range from 12% to as high as 90%. A longitudinal study emphasised that neck dissection and radiotherapy have been identified as the main risk factors in the development of secondary HNL, with the use of multiple treatment modalities also greatly increasing the risk [6].

Treatment induced HNL is commonly recognised as a contributing factor to impairment of functional domains, substantial symptom burdens and poor quality of life [8, 11]. A person with HNL will often experience skin tightness, heaviness and mobility issues in the jaw, face and neck [12, 13]. HNC patients must often accept that the effects of lymphoedema may limit their daily living activities through restrictions in functional status and altered appearance [4, 14]. There are extensive consequences of HNL but cosmetic sequelae is still regarded as the most common among HNC survivors, with 84% reporting appearance issues in a recent review [15]. It is imperative to recognise that an individual’s face and neck are vital to their self-identity and therefore the consequences of HNL can have major impact on health-related quality of life and body image [12].

Despite the prevalence and symptom burden of HNL, there remains a lack of consensus on the most effective method of diagnosis and treatment [9]. Complete Decongestive Therapy (CDT) is currently deemed the gold standard for treating HNL [16]. CDT consists of two phases with an intensive therapist led programme and maintenance phase carried out by HNC survivors [15]. Despite CDT being deemed the gold standard approach, there is limited knowledge surrounding its effectiveness [17]. Alternative treatment modalities are indicated in the literature with positive outcomes, highlighting the lack of consensus regarding the treatment of HNL. These alternative modalities have included the use of liposuction, selenium injections, kineso taping and pneumatic compression [11, 18, 19].

Although the current body of research has emphasised CDT as being the current standard of care, there remains a lack of high-level evidence-based research to direct the prevention and management of HNL [20]. This is supported by Ozdemir et al. [16] who report that most studies focus on the effects of using CDT on extremities, with only a minority based on HNL. Recent reviews emphasise that the incidence and severity of HNL can reduce with early intervention, however challenges can occur due to HNL being less visible within the early stages of development [8, 11]. The recommendation that CDT is most effective in the early stages of HNL and less effective during the chronic period, supports the use of CDT in early intervention management, as well as the potential use of alternative modalities for effective treatment [16]. It is noteworthy that HNC patients are currently referred for HNL when their symptoms are more severe, which contradicts previous research indicating that early intervention is essential for positive treatment outcomes [8, 17]. There is clearly a dearth of direction within the literature surrounding HNL management and reviews have shown the current standard of care for HNL is varied throughout the world, with the UK having no standard clinical pathway, single modality treatment or process of referral [8]. A review by Jeans et al. [9] confirms the variation within the management of HNL on mode and intensity of CDT treatment. Due to the potential symptom burden of HNL and the indication for early intervention, it is imperative that a structured and effective management intervention is available for this population [11].

Previous studies have demonstrated that HNC survivors with HNL have shown benefits when self-administering treatment in their homes after sufficient training [21]. The role of self-management would appear to be an essential component of HNL management to enable detrimental consequences of lymphoedema to be controlled [4, 22].

Despite the importance, there is limited research exploring self-management strategies within HNL management [23]. Often HNC survivors are unable to successfully implement these strategies into their lives post-treatment and manage complete adherence to programmes [3]. These challenges can prove detrimental to HNC survivor’s physical and emotional well-being, and negatively impact health-related quality of life [4]. Difficulty in implementing home management strategies could be the reason for current practices employing mostly therapist led interventions as the mainstay for the management of HNL, with a period of self-management afterwards [7]. Nonetheless, within the current body of literature there is support for self-management and emphasis on positive outcomes of improved swallowing, communication, and potential to be as beneficial as therapist-based treatments for specific individuals [7, 24].

Challenges within the different HNL management strategies in HNC survivors is evident throughout recent research. A common issue is adherence. Challenges surrounding adherence to both therapist-led CDT and self-management strategies have been highlighted, including the difficulty HNC survivors may experience in trying to find the motivation to integrate these into their daily lives after primary treatment [4]. Recommendations for further research have been made to integrate these strategies from the initial diagnosis stage to strengthen the impact on HNC survivors’ health-related quality of life [4, 25].

Compression and self-management are two main elements of HNL management when considering issues regarding adherence. Moreover, compression garments are a crucial element of the previously mentioned gold standard CDT treatment regime. Low adherence with HNC survivors is primarily due to the unique anatomy of the head and neck area and discomfort [7, 21]. The lack of adherence to self-management post-treatment is concerning as self-management compliance is reported as vital for achieving and maintaining success within HNL treatment outcomes and minimising symptom burden [26].

Although there have been studies within the field of HNL management demonstrating positive effects, there remain barriers for HNC patients in receiving and completing HNL treatment in clinical and home-based settings [27, 28]. This is a critical issue to address given the devastating effects HNL can have on body image, quality of life and functional status [11, 27]. A review by Jeans et al. [9] emphasises the importance to focus further research on effective management protocols within HNL to address the current lack of consensus on HNL management for individual HNC patients. CDT is currently the most researched management strategy overall, however the efficacy of this management strategy and alternative management on an individual basis has yet to be determined [11]. As HNL is a chronic condition it is important to recognise that HNC patient can be affected uniquely [2]. It is therefore critical to ensure that there is effective and efficient management available, tailored to individual patient’s needs by building on the current body of literature and interventions. The evident lack of consensus and adherence within the current body of literature for HNL management highlights a need for further research. This strongly argues the need to systematically collate and analytically synthesise existing HNL management strategies within intervention studies to ascertain efficacy for this population.

The aim of this systematic review is to investigate the effectiveness of HNL management strategies on function domains and health-related quality of life following treatment for HNC patients. Key objectives of the review include:

- Evaluate how effective HNL interventions are in relation to improving function related outcomes for HNC patients.
- Evaluate how effective HNL interventions are in relation to improving health-related quality of life outcomes for HNC patients.
- Identify if any specific management intervention attributes influence the main outcomes of function and health-related quality of life.

## Methods

### Study registration

This systematic review protocol has been developed following the guidelines of preferred reporting items for systematic reviews and meta-analysis protocols (PRISMA-P) [29]. The protocol has been registered on the international prospective register of systematic reviews platform (PROSPERO). The registration ID is: CRD42022378417. S1 Appendix.

### Eligibility

The types of studies included in the systematic review will follow the population, intervention, comparison, and outcome (PICO) model. Studies that will be included consist of randomised control trials, feasibility and pilot studies evaluating HNL interventions for HNC patients. Studies will be excluded if they are non-intervention, case reports or case series, and not published in English. Studies not published in English will be excluded due to lack of resources for translation. This is recognised as a limitation of the systematic review.

### Participants

Participants will be aged 18 years or more and have received a diagnosis of HNC, treatment for HNC, still undergoing treatment for HNC, and HNC survivors.

### Intervention

The review will include studies of HNL management intervention programmes such as CDT. Additionally, HNL self-management interventions which involve home-based treatments will be considered. Within chronic illnesses such as HNL, self-management can be described as the active participation of patients in the management of their condition [30]. Self-management HNL interventions can include exercise, skin care, compression, education, and massage. It is important to emphasise that this type of intervention does not exist in a vacuum but within the different contexts of individual patients and external influences [30]. Studies will also be considered of other types of HNL management interventions using different modalities, dependant on what exists in the literature.

Delivery of interventions can be through a variety of methods such as- professional-led, self-management, web-based and other electronic measures.

Studies will be excluded if there is no evident focus on HNL management for HNC or HNL patient data cannot be segregated.

### Comparison/Control

Studies that incorporate usual treatment methods, active control groups, wait-list control groups or no control group will be included. Usual treatment methods within this area of HNL management may vary given the lack of endorsed treatment pathways and guidelines [8], therefore usual care for each study will be clearly reported to enhance transparency.

Studies comparing multiple HNL management strategies will also be included.

### Outcome

The main outcome will be to investigate if there are improvements in function domains and/or health-related quality of life in HNC patients’ post-treatment, both of which are identified as chronic side-effects of HNL.

Functional outcomes that will be assessed in the studies can be defined as disease specific impairments including speech, eating, trismus, and range of motion in neck, shoulder, and jaw [12, 27, 28]. Examples of tools that are used to measure these outcome measures include the Common Terminology Criteria for Adverse Events (CTCAE) assessing trismus, ROM instruments and the Vanderbilt head and neck symptom survey for assessing functional impact [18].

Health-related quality of life outcome domains that will be evaluated in the included studies are emotional well-being, physical well-being, social engagement, body image issues, sexuality, loneliness, pain, and quality of sleep [12, 13, 22, 28]. Examples of tools used to measure these outcome measures include the European Organisation for Research and Treatment of Cancer Quality of Life Questionnaire head and neck module 43 (EORTC QLQ-H+N43) for overall HRQOL in HNC [7], Derriford Appearance Scale (DAS59) assessing psychological distress including sexuality [31], self-consciousness and body self-consciousness, and the Modified Blepharoplasty Outcome Evaluation (MBOE) assessing appearance/body image [31].

The function domains and health-related quality of life will be assessed in studies at two or more timepoints. The timeframe will involve baseline and a minimum of one additional assessment.

Studies will also be included with use of validated and non-validated assessment tools for HNL and HNC.

### Literature search and strategy

Search strategies of literature will be created using medical subject headings (MeSH) and key words in line with database specifications. The MeSH and keywords will be developed in relation to HNL management. Databases that will be used to search include Medical Literature Analysis and Retrieval System Online (MEDLINE) via Ovid and PubMed, CINAHL, Cochrane Central Register of Controlled Trials (CENTRAL) and Scopus. The reason for this selection was to ensure a broad search of literature, ensuring all relevant studies were identified. The literature search will have limits set from 2002–2022, English language, and adult human subjects only. The decision was made to extend the search limit to 20 years, therefore ensuring no studies were excluded due to the initial scoping of literature demonstrating limited research studies surrounding the management of HNL.

Examination of references in included studies provided by the search will be conducted to identify any additional studies. Citation tracking on relevant studies in combination with grey literature searching and a manual search of clinical trial websites will also be performed. This will involve websites including the United States National Library of Medicine Clinical Trials website [32] and International Clinical Trials Registry Platform [33]. The software Covidence will be implemented to export and manage the results of our search. Deduplication will be performed using this software and a bibliography of all included articles will be available to the review team.

Specific search strategies for the systematic review will be developed in collaboration with a Life and Health Sciences subject expert Librarian. Searches will be carried out using the truncation of the terms “lymphoedema”, “edema”, “swelling”, “head and neck”, “head and neck neoplasms”, “head and neck cancer”, “head and neck malignancy”. After one database search is finalised, it will be adapted to the other database criteria according to the MeSH and keyword truncation guidelines. Searches will be updated in the later stages of the systematic review to ensure all eligible studies are included. An example of one planned search strategy performed in June 2023 is provided in the supplementary information titled S2 Appendix.

### Data extraction

Covidence will be used to export and manage search results. Two methods will be incorporated to screen study results. Firstly, the title and abstract will be screened in accordance with eligibility criteria for relevance. Secondly, full texts of potentially eligible trials will be obtained and assessed for inclusion. Any studies that are excluded in line with eligibility criteria will be recorded and justified. Two researchers will independently carry out the review process for screening and eligibility assessment to ensure consistency. Potential discrepancies will be resolved through consultation with a third researcher.

A standardised coding form will be created to enable analytical extraction of data and piloted to ensure the following items are collected from the studies:

- Study details such as title, author, year of publication and country of origin.
- Study characteristics to include the sample number, grouping and design methods.
- Patient demographic and clinical characteristics to include age, sex and ethnicity.
- Clinical characteristics and treatment information for HNC.
- Characteristics of intervention including type of HNL management, mode of intervention delivery, duration of study, baseline severity of HNL and intervention treatment information.
- Details of control and comparison groups.
- Outcomes for HNL assessment of function domains and health-related quality of life.
- Intervention effectiveness and quality.
- Evaluation of participant satisfaction and adherence.

### Data evaluation

The potential risk of bias and quality for each study included in this review will be assessed using the Cochrane Handbook of Systematic Reviews of Intervention risk of bias tool [34] and quality appraisal of the evidence for using the Grading of recommendations, Assessment, Development and Evaluation (GRADE) tool and GRADEpro software [34]. To enhance rigour, a second author (CS) will independently complete GRADE for 10% of the included studies. The GRADE assessment will include 5 domains including risk of bias, inconsistency of results, indirectness, imprecision, and publication bias. The levels for certainty of evidence will be rated as high, moderate, low, and very low. The risk of bias will utilise the revised risk of bias tool RoB2 for randomised trials and ROBINS-1 tool for non-randomised studies. Signalling questions will be followed using the RoB2 for randomised control trials to determine the risk of bias from study information and will be expressed as low, high or of some concern. If there is insufficient information available, then the risk of bias will be determined as unclear. The corresponding author for any study with discrepancies regarding risk of bias will be contacted to seek clarity on their study protocol and methodology, if appropriate.

The process of determining risk of bias will be performed by two researchers. One reviewer will review the studies independently to assess risk of bias. The second researcher will then review a selection of the studies to ensure validity and accuracy. Potential disagreements will be resolved by discussion initially and then by an additional researcher. Data surrounding risk of bias will be presented narratively using the GRADEpro software [35].

### Data synthesis

The review will examine the effects of HNL management on function domains and health-related quality of life in HNC patients. A meta-analysis will be incorporated if there are sufficient studies that are homogenous in their design, intervention features and outcome measures. If deemed appropriate, this meta-analysis will be performed using a random effects model with the statistical software RevMan 5 to provide an estimate of intervention effect [36]. Estimated standard mean differences will be pooled with 95% confidence intervals calculated for any outcomes that are continuous [37]. Dichotomous outcomes will involve analysis using a risk ratio and 95% confidence intervals [37].

If there is insufficient homogeneity in interventions and outcomes between studies in the review, then a systematic narrative of the study findings will be conducted which will follow the information provided in the Cochrane Handbook for Systematic Reviews of Interventions [34]. Initially, quantitative data will be extracted and transformed into text to produce a data extraction table, including information on author(s), year, country, title, aims, research design, sample characteristics, outcome measures, main findings, and barriers/facilitators of the intervention. This will enable a preliminary systematic and comprehensive synthesis of the results of each study, highlighting important characteristics of the studies, alongside important similarities, or differences (for example, in study design, populations, intervention components, outcome measures or other elements). Relationships within and between studies will be explored, with exploration of patterns in the data, considering the results of studies of different design, and different forms of intervention characteristics, implementation, or delivery. These findings will be reported thematical. The GRADE guidelines and GRADEpro software will be utilised to assess the methodological quality of the included studies. This will form the basis for reporting on the quality of the evidence in the review. If appropriate, the robustness of results will be assessed through completion of a sensitivity analysis, and publication bias will be assessed with use of funnel plots.

### Sub-group analysis

Sub-group analysis will be conducted if there is sufficient data available. Included studies will be grouped depending on intervention treatment type, mode of intervention delivery, length of follow up and control groups. A separate meta-analysis will then be performed based on the groupings listed above to calculate the pooled effect sizes of the HNL management strategies.

### Public and patient involvement

There will be no patient and public involvement (PPI) in this systematic review.

### Ethics

There will be no ethical requirements for this systematic review as there is no PPI. The results of the review will be disseminated in conferences and published in a peer review journal.

## Discussion

Systematic review protocols have been highlighted as a critical step in the review process as they ensure transparency and ensure bias is minimised [34]. The protocol has been designed to enable methodological transparency and enhance understanding of the proposed systematic review process. We have developed the systematic protocol in accordance with the guidelines provided by the PRISMA-P checklist, as evidenced in the supplementary information titled S3 Appendix. To the best of our knowledge, this is the only systematic review to evaluate the efficacy of HNL management strategies and the effect on functional domains and health-related quality of life in HNC patients after treatment completion. There is a dearth of evidence-based research surrounding HNL, with the majority of research completed within breast cancer and other extremities [6]. Although there have been studies within the field of HNL management demonstrating positive effects, there remain barriers for HNC patients in receiving and completing HNL treatment in clinical and home-based settings [27, 28]. This is a critical issue to address considering the devastating effects HNL can have on patient’s body image, health-related quality of life and functional domains [11, 27]. These devasting effects can lead to social isolation and social avoidance among this cancer population [38]. The seriousness of this condition is emphasised by the estimation that more than 50% of HNC patients experience HNL [8].

This systematic review will enhance the awareness and understanding regarding the effectiveness of individual HNL strategies and provide a basis for the development of an intervention-based study, using public and patient involvement to improve function and health-related quality of life in HNC patients’ post-treatment [39]. Such an intervention will be fundamental to positively impacting HNC patients’ health-related quality of life after treatment. It is predicted that the results of this review will therefore significantly build on the existing knowledge around HNL management.

## Supporting information

S1 Appendix PROSPERO international prospective register of systematic reviews. (PDF)

S2 Appendix Ovid MEDLINE search strategy. (DOCX)

S3 Appendix PRISMA-P checklist. (DOCX)
