# Supplementary material for: Evaluating the effects of lymphoedema management strategies on functional status and health-related quality of life following treatment for head and neck cancer: Protocol for a systematic review
Source: PLoS One. 2024 Feb 2;19(2):e0297757. doi: 10.1371/journal.pone.0297757 (PMC10836692; doi:10.1371/journal.pone.0297757)
Supplement: S1 Appendix — (PDF) [file pone.0297757.s001.pdf]

Evaluating the effects of lymphoedema management strategies on functional status and health-related quality of life following treatment for head and neck cancer patients

Citation

Lauren Mullan, Cherith Semple, Nicole Blackburn, Jill Lorimer. Evaluating the effects of lymphoedema management strategies on functional status and health-related quality of life following treatment for head and neck cancer patients. PROSPERO 2022 CRD42022378417 Available from:
https://www.crd.york.ac.uk/prospero/display_record.php?ID=CRD42022378417

Review question

The aim of this current systematic review is to investigate the effectiveness of head and neck lymphoedema management strategies on function domains and health-related quality of life following treatment for head and neck cancer patients.

Searches

The search strategy will incorporate the use of the following electronic databases: Ovid MEDLINE, PubMed, CINAHL, the Cochrane Central Register of Controlled Trials (CENTRAL) and Scopus. Databases will be searched from time of inception to November 2022.

Search strategies of literature will be created by the use of medical subject headings (MeSH) and key words in line with database specifications. The MeSH and keywords will be developed in relation to head and neck lymphoedema management.

The literature search will have limits of English language and adult human subjects.

Examination of references in included studies provided by the search will be conducted to identify any additional studies. Citation tracking on relevant studies in combination with grey literature searching and a manual search of clinical trial websites will also be performed.

Types of study to be included

Study designs of randomised control trials, feasibility and pilot studies evaluating head and neck lymphoedema interventions for head and neck cancer patients will be included.

Studies will be excluded if they are non-intervention and not published in English.

Condition or domain being studied

The management of head and neck lymphoedema in head and neck cancer patients.

Participants/population

Participants are aged 18 years or more and have received a diagnosis of head and neck cancer, treatment for head and neck cancer, still undergoing treatment for head and neck cancer, and head and neck cancer survivors.

Intervention(s), exposure(s)

Inclusion criteria for the review includes studies of head and neck lymphoedema management intervention programs such as Complete Decongestive Therapy.

Additionally, head and neck lymphoedema self-management interventions will be considered. Studies will also be considered of other types of head and neck lymphoedema management interventions using different modalities, dependent on what exists in the literature.

Delivery of interventions can be through a variety of methods such as in person, web-based and other electronic measures.

Studies will be excluded if there is no evident focus on head and neck lymphoedema management for head and neck cancer or head and neck lymphoedema patient data cannot be segregated.

Comparator(s)/control

Studies that incorporate usual treatment methods, active control groups, wait-list control groups or no control group will be included.

Studies comparing multiple head and neck lymphoedema management strategies will also be included.

Main outcome(s)

The main outcome will be to investigate if there are improvements in function domains and/or health-related quality of life in head and neck cancer patients' post-treatment.

Functional domain outcomes that will be assessed in the studies can be defined as disease specific impairments including speech, eating, trismus and range of motion in neck, shoulder and jaw.

Health-related quality of life outcome domains that will be evaluated in the included studies are emotional well-being, physical well-being, social engagement, body image issues, sexuality, loneliness, pain and quality of sleep.

The function domains and health-related quality of life will be assessed in studies at two or more timepoints. The timeframe will involve baseline and a minimum of one additional assessment.

Studies will be included with use of validated and non-validated assessment tools for head and neck lymphoedema and head and neck cancer.

Additional outcome(s)

None.

Data extraction (selection and coding)

Covidence will be used to export and manage search results. Two methods will be incorporated to screen study results. Firstly, the title and abstract will be screened in accordance to eligibility criteria for relevance. Secondly, full texts of potentially eligible trials will be obtained and assessed for inclusion. Any studies that are excluded in line with eligibility criteria will be recorded and justified.

Two researchers will independently carry out the review process for screening and eligibility assessment to ensure consistency. Potential discrepancies will be resolved through consultation with a third researcher.

A standardised coding form will be created to enable analytical extraction of data and piloted to ensure the following items are collected from the studies:

1. Study details such as title, author, year of publication, country of origin and journal type.

2. Study characteristics to include the sample number, grouping and design methods.

3. Patient demographic and clinical characteristics to include age, sex and ethnicity.

4. Clinical characteristics and treatment information for HNC.

5. Characteristics of intervention including type of HNL management, mode of intervention delivery, duration of study, baseline severity of HNL and intervention treatment information.

6. Outcomes for HNL assessment of function domains and health-related quality of life.

7. Intervention effectiveness and quality.

8. Evaluation of participant satisfaction and adherence.

Risk of bias (quality) assessment

The potential risk of bias will be assessed for each study in this review using the revised risk of bias tool RoB2 for randomised trials and ROBINS-I tool for non-randomised studies. The domains that will be covered include generation of allocation sequence, concealment of allocation, blinding of intervention, outcome handling and measurement of incomplete data and selective outcome reporting. Decisions regarding potential risk of bias for each of the various domains will be established from study information and determined as either low or high risk. If there is insufficient information available, then the risk of bias will be determined as unclear. The key investigator will be contacted for further information if appropriate.

Determining the risk of bias will be performed by two researchers. One reviewer will review the studies independently to assess risk of bias. The second researcher will then review a selection of the studies to ensure validity and accuracy. Potential disagreements will be resolved by discussion and then by an additional researcher. Data surrounding risk of bias will be presented narratively and graphical representation produced in the RevMan 5.1 software (Review Manager).

Each paper will be assessed for quality using the Critical Appraisal Skills Programme (CASP) tool.

Strategy for data synthesis

The review will examine the effects of head and neck lymphoedema management on function domains and health-related quality of life in head and neck cancer patients. A meta-analysis will be incorporated if there are sufficient studies that are homogenous in their design and outcomes. This meta-analysis will be performed using the statistical software RevMan 5.1 (Review Manager).

If there is insufficient homogeneity in interventions and outcomes between studies in the review, then a systematic narrative of the study findings will be conducted. The synthesis will be provided through information in text and table format to present a summary and explanation of the findings of included studies in the review.

Analysis of subgroups or subsets

Sub-group analysis will be conducted if there is sufficient data available. Included studies will be grouped depending on intervention treatment type, mode of intervention delivery, length of follow up and control groups. A separate meta-analysis will be performed based on the groupings listed above to calculate the pooled effect sizes of the head and neck lymphoedema management strategies.

Contact details for further information

Lauren Mullan

Mullan-L1@ulster.ac.uk

Organisational affiliation of the review

University of Ulster

www.ulster.ac.uk

Review team members and their organisational affiliations

Ms Lauren Mullan. Ulster University

Dr Cherith Semple. Ulster University

Dr Nicole Blackburn. Ulster University

Ms Jill Lorimer. Belfast Health and Social Care Trust

Type and method of review

Intervention, Systematic review

Anticipated or actual start date

28 November 2022

Anticipated completion date

30 October 2023

Funding sources/sponsors

PhD studentship funded by the Department for the Economy

Conflicts of interest

Language

English

Country

Northern Ireland

Stage of review

Review Ongoing

Subject index terms status

Subject indexing assigned by CRD

Subject index terms

Functional Status; Head and Neck Neoplasms; Humans; Lymphedema; Quality of Life; Surveys and Questionnaires

Date of registration in PROSPERO

28 November 2022

Date of first submission

24 November 2022

Stage of review at time of this submission

The review has not started

Stage	Started	Completed
Preliminary searches	No	No
Piloting of the study selection process	No	No
Formal screening of search results against eligibility criteria	No	No
Data extraction	No	No
Risk of bias (quality) assessment	No	No
Data analysis	No	No

The record owner confirms that the information they have supplied for this submission is accurate and complete and they understand that deliberate provision of inaccurate information or omission of data may be construed as scientific misconduct.

The record owner confirms that they will update the status of the review when it is completed and will add publication details in due course.

Versions

28 November 2022
