# Supplementary material for: Evaluating the effects of lymphoedema management strategies on functional status and health-related quality of life following treatment for head and neck cancer: Protocol for a systematic review
Source: PLoS One. 2024 Feb 2;19(2):e0297757. doi: 10.1371/journal.pone.0297757 (PMC10836692; doi:10.1371/journal.pone.0297757)
Supplement: S2 Appendix — (DOCX) [file pone.0297757.s002.docx]

| Database: Ovid MEDLINE(R) ALL <1946 to July 03, 2023>  Search Strategy:  1 Lymphedema/ (10582)  2 (lymphedema or lymphoedema or edema or swelling).mp. [mp=title, book title, abstract, original title, name of substance word, subject heading word, floating sub-heading word, keyword heading word, organism supplementary concept word, protocol supplementary concept word, rare disease supplementary concept word, unique identifier, synonyms, population supplementary concept word, anatomy supplementary concept word] (283604)  3 1 or 2 (283604)  4 “Head and Neck Neoplasms"/ (64757)  5 ("head and neck cancer*" or "head and neck malignancy" or "head and neck neoplasm*" or laryngeal cancer or oral cancer or head cancer or neck cancer).mp. [mp=title, book title, abstract, original title, name of substance word, subject heading word, floating sub-heading word, keyword heading word, organism supplementary concept word, protocol supplementary concept word, rare disease supplementary concept word, unique identifier, synonyms, population supplementary concept word, anatomy supplementary concept word] (99681)  6 4 or 5 (99681)  7 3 and 6 (1101)  8 limit 7 to (english language and yr="2002 -Current" and "all adult (19 plus years)") (399) |
| --- |

S2: Example of search strategy carried out in Ovid MEDLINE.
